# Analysis of Inertial Measurement Unit Data for an AI-Based Physical Function Assessment System Using In-Clinic-like Movements

**DOI:** 10.3390/bioengineering11121232

**Published:** 2024-12-05

**Authors:** Nobuji Kouno, Satoshi Takahashi, Ken Takasawa, Masaaki Komatsu, Naoaki Ishiguro, Katsuji Takeda, Ayumu Matsuoka, Maiko Fujimori, Kazuki Yokoyama, Shun Yamamoto, Yoshitaka Honma, Ken Kato, Kazutaka Obama, Ryuji Hamamoto

**Affiliations:** 1 Division of Medical AI Research and Development, National Cancer Center Research Institute, 5-1-1 Tsukiji, Chuo-ku, Tokyo 104-0045, Japan; satoshi.takahashi.fy@riken.jp (S.T.); ken.takasawa@riken.jp (K.T.); maskomat@ncc.go.jp (M.K.); 2 Cancer Translational Research Team, RIKEN Center for Advanced Intelligence Project, 1-4-1 Nihonbashi, Chuo-ku, Tokyo 103-0027, Japan; naoaki.ishiguro@riken.jp (N.I.); katsuji.takeda@riken.jp (K.T.); 3 Department of Surgery, Graduate School of Medicine, Kyoto University, 54 Shogoin-kawahara-cho, Sakyo-ku, Kyoto 606-8507, Japan; kobama@kuhp.kyoto-u.ac.jp; 4 Division of Survivorship Research, National Cancer Center Institute for Cancer Control, 5-1-1 Tsukiji, Chuo-ku, Tokyo 104-0045, Japan; aymatsuo@ncc.go.jp (A.M.); mfujimor@ncc.go.jp (M.F.); 5 Department of Head and Neck, Esophageal Medical Oncology, National Cancer Center Hospital, 5-1-1 Tsukiji, Chuo-ku, Tokyo 104-0045, Japan; kazyokoy@ncc.go.jp (K.Y.); shuyamam@ncc.go.jp (S.Y.); yohonma@ncc.go.jp (Y.H.); kenkato@ncc.go.jp (K.K.)

**Keywords:** objective physical function assessment, timed up and go test, short physical performance battery, inertial measurement unit, patients with cancer, artificial intelligence

## Abstract

Assessing objective physical function in patients with cancer is crucial for evaluating their ability to tolerate invasive treatments. Current assessment methods, such as the timed up and go (TUG) test and the short physical performance battery, tend to require additional resources and time, limiting their practicality in routine clinical practice. To address these challenges, we developed a system to assess physical function based on movements observed during clinical consultations and aimed to explore relevant features from inertial measurement unit data collected during those movements. As for the flow of the research, we first collected inertial measurement unit data from 61 patients with cancer while they replicated a series of movements in a consultation room. We then conducted correlation analyses to identify keypoints of focus and developed machine learning models to predict the TUG test outcomes using the extracted features. Regarding results, pelvic velocity variability (PVV) was identified using Lasso regression. A linear regression model using PVV as the input variable achieved a mean absolute error of 1.322 s and a correlation of 0.713 with the measured TUG results during five-fold cross-validation. Higher PVV correlated with shorter TUG test results. These findings provide a foundation for the development of an artificial intelligence-based physical function assessment system that operates without the need for additional resources.

## 1. Introduction

The global incidence of patients with cancer is on the rise [1], particularly as the population ages, leading to an increasing number of older patients with cancer [2,3]. Despite the remarkable advancements in surgery and chemotherapy, cancer treatment inevitably remains invasive, even when therapeutic benefits are evident. For older patients, chronological age alone does not adequately reflect their ability to tolerate invasive treatments [4], making a thorough assessment of each patient’s physical function crucial for informed treatment decisions [5].

For clinicians in oncology, the Eastern Cooperative Oncology Group Performance Status (PS) is the most widely used metric for evaluating physical function [6,7,8]. PS evaluates activities of daily living on a 5-point scale from 0 to 4 [9] and is used to assess eligibility for invasive cancer treatments, such as surgery and chemotherapy, and as a criterion in many clinical trials. However, PS has some limitations. Patients with the same PS may have varying backgrounds, indicating a lack of objectivity [10]. Additionally, PS results can vary among clinicians [11], leading to discrepancies in perceptions within healthcare teams and among patients. As treatment progresses, patients’ physical function may change due to the treatment or its side effects, but the PS does not effectively capture these subtle changes [10]. Despite its simplicity, PS documentation in cancer care remains notably low [12].

More objective assessments, such as the short physical performance battery (SPPB) and timed up and go (TUG) tests, are available. The SPPB consists of three subdomains on a 12-point scale, balance, walking, and standing [13,14,15], while the TUG test evaluates the total time taken to perform a series of actions: standing up, walking, turning around, and sitting down [16,17,18]. Both assessments correlate with clinical outcomes, such as mortality [19], postoperative complications [5], and chemotherapy-related events [20]. While these assessments are more objective than PS assessments, barriers exist to their implementation in routine clinical practice [21]. They often require dedicated spaces and personnel, as well as additional time for both patients and healthcare providers. At least one healthcare professional is required to instruct, monitor, and time the assessments, and further assistance may be necessary for fall prevention or balance support, creating challenges for their routine implementation [21].

Several studies have investigated the use of digital technologies to alleviate these resource constraints. For example, Soubra et al. developed a system using a Doppler radar attached to a chair to automatically detect phases of standing, sitting, and walking during the TUG, automating time measurement [22]. Hellmers et al. achieved a correlation coefficient of 0.97 with manual measurements by having patients with Parkinson’s disease perform TUG while wearing an accelerometer [23]. Duncan et al. created a system using multiple remote cameras to automatically detect and measure movements during the SPPB and TUG tests [24], while Savoie et al. developed a system that uses depth-sensing cameras to automate TUG measurements [25]. However, these systems do not fully address the additional time required for SPPB or TUG assessments, and the use of additional equipment may impose a burden on resource-limited hospitals.

Alternatively, several studies have focused on predicting the results of objective physical function assessments without actually conducting the assessments using historical sensor data, medical records, or other physical function assessments. Sasani et al. developed a decision tree model to predict TUG results using questionnaire-based functional assessments with a cutoff value of 10 s in older adults [26]. Hasegawa et al. developed a binary classification model for the SPPB with a cutoff value of 6 points for men and 8 points for women based on medical records, grip strength, and body measurements [27]. Kraus et al. created several machine learning regression models to predict TUG results using blood test data, grip strength, and patient-reported data [28]. Bloomfield et al. developed a classification model to predict the differences in TUG test results before and after knee arthroplasty using spatiotemporal parameters from inertial measurement units (IMUs) [29]. Similarly, Polus et al. predicted TUG results 6 weeks postoperatively with a cutoff value of 14 s for patients with hip arthroplasty using IMU data collected at preoperative and 2-week postoperative periods [30]. However, these studies did not fully meet the need for timely assessments during unexpected declines in physical function.

Recently, the use of artificial intelligence (AI) in the medical field has been progressing, and it is being used in various fields, such as medical image analysis, including radiological image analysis and endoscopic image analysis, omics analysis, and the analysis of medical information using natural language processing technology [31,32,33,34,35,36,37,38,39]. Under these circumstances, utilizing AI for routine physical function assessments in clinical practice is an important strategy. With the aim of overcoming the above-mentioned issues, we conceptualized an AI-based approach that predicts objective physical function assessments from videos capturing movements like walking, sitting, and standing in confined spaces such as a consultation room. These videos can be recorded using a stationary camera set in the consulting room, requiring no additional time or resources during consultation, thus overcoming resource-related barriers. Additionally, this system facilitates longitudinal physical function assessment over time rather than at a single time point.

However, the development of AI models based on video input presents the “black box” problem [40,41], where the opaque nature of these models may complicate their clinical implementation, potentially impacting healthcare decision-making. Furthermore, capturing the entire body in the limited space of a consultation room poses challenges, and it is unclear which body parts should be prioritized to adequately infer physical function from routine movements.

As a first step toward developing an AI-based physical function assessment system using videos, our study aimed to explore relevant features from IMU data collected during movements that replicate a series of actions in a consultation room. These features will serve as the foundation for developing an AI model to predict objective physical function assessments.

### Research Gaps and Contributions

To address the limitations and challenges outlined above, we focused on the following key research gaps and contributions:

Research gap 1: Existing objective physical function assessment tools, such as the SPPB and TUG, are resource-intensive, limiting their routine use in clinical practice.
Contribution 1: We propose an AI-based approach that minimizes the need for additional space, time, or personnel by leveraging features derived from routine movements.
Research gap 2: Previous studies using IMU or sensor data to assess physical function have not identified the most informative features or body regions to simplify sensor setups.
Contribution 2: We identified and evaluated key features and body regions (e.g., the pelvis) relevant for accurate predictions, paving the way for simplified systems that require fewer sensors.
Research gap 3: Current approaches often fail to provide timely and longitudinal assessments of physical function.
Contribution 3: Our conceptual AI-based approach enables seamless and repeated assessments of physical function over time, providing opportunities for the early detection of decline or changes in physical function.
Research gap 4: AI-based systems in healthcare often face the “black box” problem, which limits clinical implementation.
Contribution 4: By identifying interpretable features such as pelvic velocity variability, this study lays the foundation for creating transparent AI models that clinicians can trust.

The remainder of this paper is organized as follows: Section 2 describes the methodology, including data collection and model development. Section 3 presents the results, including the model performance metrics. Section 4 discusses the results of previous studies and their limitations. Section 5 outlines future directions, focusing on expanding target populations, refining the model, and integrating longitudinal assessments. Section 6 concludes the paper with a summary of key findings and implications.

## 2. Materials and Methods

### 2.1. Patient Recruitment and Logistics

We recruited patients aged 18 years or older with a PS of 0–2 who were admitted for chemotherapy in the Department of Head and Neck, Esophageal Medical Oncology at the National Cancer Center Hospital between May 2023 and March 2024. The chemotherapy regimens encompassed chemoradiotherapy, neoadjuvant or induction chemotherapy, and palliative chemotherapy. Patients were excluded if they were unable to make decisions due to dementia, posed a risk of contact or airborne infections, or were deemed unsuitable for participation by their attending physician. Additionally, patients whose chemotherapy admissions occurred on weekends or days when the conference room for objective physical function assessment was unavailable were excluded.

The patients were recruited during their hospital stay for chemotherapy. After providing written informed consent, objective physical function assessments, including the SPPB and TUG, were mostly conducted on the first day of their next chemotherapy session. During this time, the patients performed movements that replicated a series of actions in a consultation room. The assessments were performed in a conference room at the National Cancer Center Hospital. This study was approved by the Institutional Review Board of the National Cancer Center (protocol code: 2022-288).

### 2.2. Data Collection

#### 2.2.1. Objective Physical Functional Assessment

**Figure 1:**
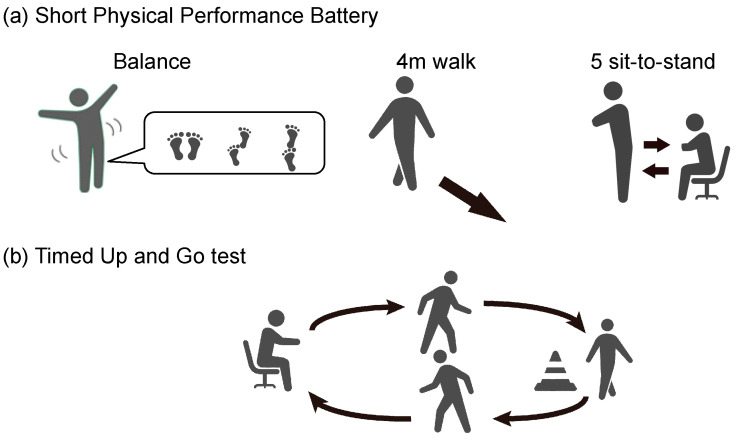
Schematic representation and detailed scoring of the short physical performance battery (SPPB) and timed up and go (TUG) test. (**a**) SPPB consists of 3 components. Balance (maximum 4 points): closed leg standing for 10 s (+1 point), semi-tandem standing for 10 s (+1 point), tandem standing for 3 to less than 10 s (+1 point), and tandem standing for 10 s (+2 points). A 4 m walk (maximum 4 points): less than 4.82 s (+4 points), 4.82–6.2 s (+3 points), 6.2–8.7 s (+2 points), and more than 8.70 s (+1 point). A 5 sit-to-stand test (maximum 4 points): less than 11.2 s (+4 points), 11.2–13.7 s (+3 points), 13.7–17 s (+2 points), 17 s or more (+1 point), and 60 s or more (0 points). (**b**) In TUG, the participant starts seated, then stands up at a signal, walks 3 m to a cone, turns around, and returns to sit in the chair, with the total time measured.

SPPB and TUG tests were conducted for each participant. The SPPB consists of three components with a total possible score of 12 points [10]: balance assessment, a 4 m walk test, and a 5-time sit-to-stand test. Specifically, four points were allocated for the ability to maintain balance in the side-by-side, semi-tandem, and tandem stances for 10 s each; four points for the time taken to walk 4 m; and four points for the time required to complete five chair stands. The TUG measures the time taken to stand up from a chair, walk 3 m, turn, walk back 3 m, and then sit again in the chair [11]. Details of the SPPB and TUG tests are shown in Figure 1.

The assessment process was supervised by an occupational therapist involved in the study, and at least two researchers conducted the assessments. To ensure patient safety, a waist belt with handles was used to assist patients in case of falls.

#### 2.2.2. Movements Replicating Series of Actions in a Consultation Room

**Figure 2:**
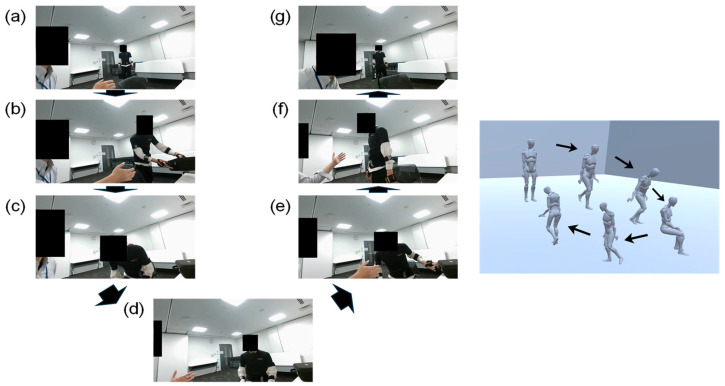
Movements replicating a series of actions in a consultation room. Movements were performed in a 1.2 m × 2.5 m space, simulating a typical consultation room. (Left person) The researcher in the role of the doctor; (Right person) the participant as the patient. (**a**–**c**) The participant walks approximately 2.5 m, places the bag on the desk, and then sits down in a chair while instructed by the researcher. (**d**) The participant engages in a brief conversation with the researcher. While sitting, the participant does not receive any instructions regarding behavior. The participant naturally nods his or her head and places his or her hands on his or her knees. (**e**–**g**) The participant stands up, picks up the bag, puts the chair back in place, and then walks back for about 2.5 m according to the researcher’s verbal instructions. Throughout all movements, the researcher does not assist the participant.

One researcher acted as the physician sitting beside a desk. Initially, each participant began walking when their names were called. Following the researcher’s instructions, participants placed their bags on the desk and sat in a chair. After a brief conversation, participants stood up, picked up their bags, and then walked back to the starting point. These movements were designed to mimic a typical series of actions observed in consultation rooms at Japanese medical institutions (Figure 2 and Supplementary Video S1). IMU data were recorded during these movements.

#### 2.2.3. IMU Data Collection and Exportation

**Figure 3:**
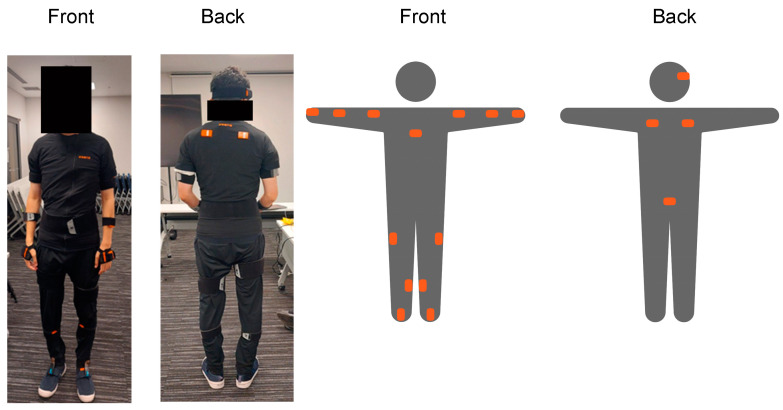
IMU locations on the body: Each IMU is placed at key body locations to accurately measure motion. (Front) IMUs are attached to the bilateral upper arms, bilateral forearms, bilateral hands, sternum, bilateral upper legs, bilateral lower legs, and bilateral feet using straps and a special t-shirt. (Back) IMUs are attached to the head, bilateral shoulders, and pelvis. IMU: inertial measurement unit.

We utilized the Xsens Awinda system (Movella, Henderson, NV, USA) for the IMUs. The Xsens Awinda consists of 17 IMUs attached to various points on the body, enabling real-time measurement of movements in a 3D space. The IMU locations are shown in Figure 3. Each participant with the IMUs attached performed movements that replicated a series of actions in a consultation room, as previously described. The IMU data were collected at 60 frames per second and recorded as time-series data for position, velocity, and acceleration at 23 keypoints. The data were reprocessed using the default HD reprocessing function in Xsens Awinda, and the time-series data at 23 keypoints were exported as CSV files and analyzed using Python 3.9.13.

### 2.3. Data Preprocessing, Label Setting, Focused Keypoints, and Feature Engineering

The time-series data were smoothed using a moving average with a window size of 5, equivalent to 5/60 s. Initially, we evaluated the distribution of the objective physical function assessment results to determine the appropriateness of the prediction label. Next, we analyzed the correlation between the selected label (SPPB and/or TUG) and the statistical features derived from the velocities of the 23 keypoints to identify the keypoint of focus. Subsequently, we engineered the features of the selected keypoints.

### 2.4. Feature Selection and Model Comparison

We performed a five-fold cross-validation using the engineered features from the focused keypoint. Feature extraction was conducted on each training set, followed by model comparison on the corresponding validation sets, using Python 3.9.13. Randomness was incorporated into Lasso regression and cross-validation through model parameter initialization and data splitting. The candidate models included linear regression (LR), random forest (RF), support vector machine (SVM), and extreme gradient boosting (XGB). After frequency analysis of the selected features in each fold, we determined the final features for the model.

## 3. Results

### 3.1. CONSORTDiagram and Cohort Profile

**Figure 4:**
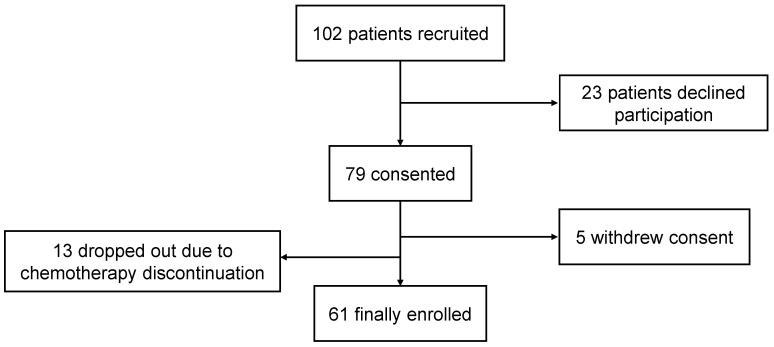
CONSORT diagram.

A total of 102 patients were initially recruited for this study. Of these, 23 declined to participate, resulting in 79 patients providing informed consent. Common reasons for declining were that patients did not have the physical or mental capacity, or that they were not interested in our research. Five patients subsequently withdrew their consent mainly due to self-reported physical function worsening, reducing the cohort to seventy-four. Additionally, 13 patients dropped out due to discontinuation of chemotherapy. Ultimately, 61 patients were the analysis subjects (Figure 4).

The cohort characteristics of the 61 patients are summarized in Table 1. The median age of the cohort was 71 years, comprising 48 males and 13 females. In terms of PS, 33 patients had a PS of 0, while the remaining 28 had a PS of 1. Regarding cancer type, 44 patients were diagnosed with esophageal cancer, 13 with head and neck cancer, 2 with gastroesophageal junction cancer, and 2 with other cancer types. The distribution of the cancer stages was as follows: stage I, 10 participants; stage II, 3 participants; stage III, 22 participants; and stage IV, 17 participants. Nine patients experienced cancer recurrence. Regarding chemotherapy type, 44 patients underwent induction /neoadjuvant chemotherapy, 11 received chemoradiotherapy, and 6 received palliative chemotherapy.

### 3.2. Label Selection and Task Setting Based on Distribution of the Results of Objective Physical Function Assessment

**Figure 5:**
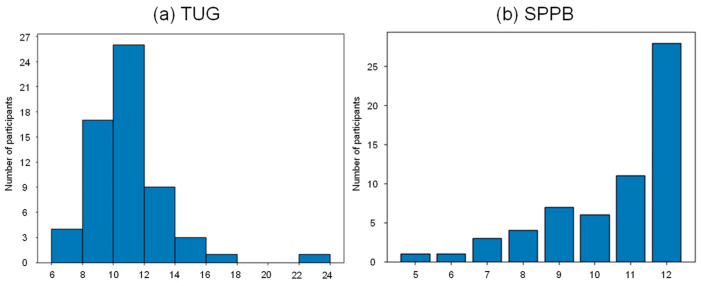
Distribution of the results of objective physical function assessment. (**a**) The TUG scores show a nearly normal distribution. (**b**) SPPB scores between 0 and 4 are missing.

Figure 5 illustrates the distribution of the objective physical function assessment results. The SPPB scores ranged from 0 to 12; however, scores between 0 and 4 were missing. Additionally, the distribution was imbalanced, with nearly half the participants scoring 12. In contrast, the TUG test results showed a nearly normal distribution, ranging from as low as 6 s to over 20 s. Therefore, we selected the TUG test as the prediction label, with the model task set as regression.

### 3.3. Identification of the Most Informative Sensor Location

**Figure 6:**
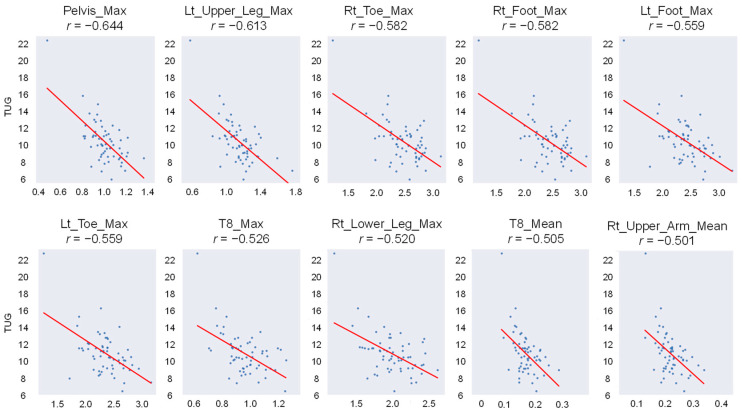
Top 10 features in the xy-plane with the highest absolute values of Pearson’s correlation coefficient (r) in relation to the results of TUG. The red lines represent the linear regression lines, highlighting the relationship between the TUG scores and the respective features. Lt: left, Rt: right, T8: Thoracic 8th, Max: maximum.

To identify the keypoint most suitable for further analysis, we calculated the maximum, minimum, and mean velocities in the xy-plane for the 23 keypoints derived from the 17 IMUs. The top ten features in the xy-plane with the highest absolute values of Pearson’s correlation coefficient (r) in relation to the results of the TUG test are shown in Figure 6. The maximum velocity of the pelvis keypoint exhibited the strongest correlation, with r = −0.644. Other keypoints with high absolute values were located in the lower limbs and along the spinal axis. Based on these findings, the pelvis keypoint was selected for further feature engineering and analysis.

### 3.4. Feature Engineering from the Pelvis Keypoint

For each case, we calculated statistical values based on velocity and acceleration data of the pelvis keypoint: maximum, minimum, mean, and standard deviation. The calculations were performed across different directions, including the z-axis, xy-plane, and xyz-direction.

**Figure 7:**
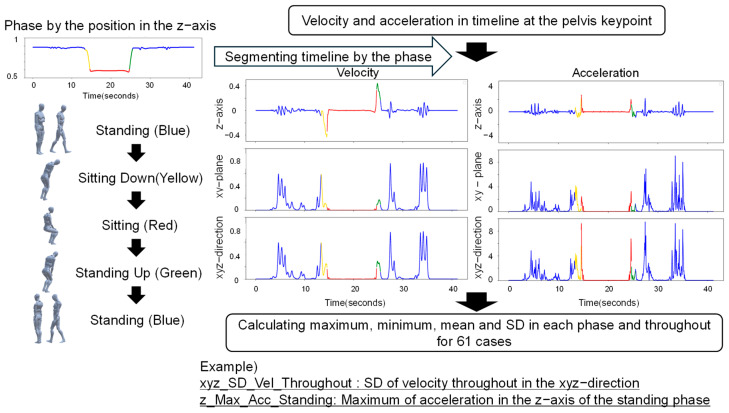
Feature engineering from the pelvis keypoint, segmented by the phase of the position in the z-axis. Examples of feature names are presented in Table 2. Vel: velocity; Acc: acceleration.

Additionally, the movement data were divided into four phases based on the z-axis position: standing, sitting down, sitting, and standing up. The four statistical values mentioned above were calculated for each phase and across the entire timeline. In total, 120 features were engineered from the pelvis keypoint data for 61 cases (Figure 7).

### 3.5. Feature Selection with Frequency Analysis and Model Comparison

We performed Lasso regression with five-fold cross-validation to reduce the number of features to a maximum of three for each training set. We used coefficients where the number of non-zero coefficients first approached but did not exceed three. In the Lasso regression, we varied the alpha and retained the coefficients, where the number of non-zero coefficients was just below three. Among these, variables with the largest absolute coefficients were selected. The selected variables from each fold were used to train models using LR, RF, SVM, and XGB, with their performances compared on the corresponding validation set.

Frequency analysis of the selected features revealed that the standard deviation (SD) of velocity in the xyz-direction throughout the timeline (referred to as Pelvis Velocity Variability, PVV) was selected in all folds (Table 2). To assess multicollinearity among the three variables in each fold, we calculated the variance inflation factor (VIF). The results showed that nearly all features had a VIF greater than 10, indicating strong multicollinearity between the variables.

**Figure 8:**
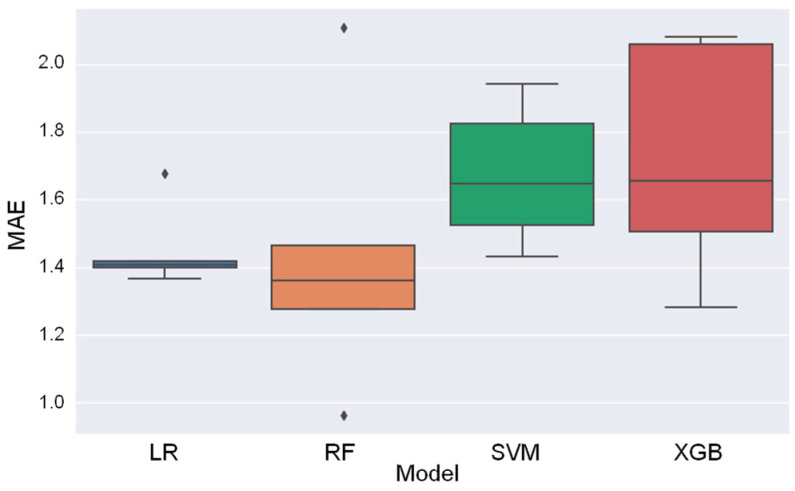
Distribution of MAE of the validation set results of five-fold cross-validation from four models. MAE: mean absolute error, LR: linear regression, RF: random forest, SVM: support vector machine, XGB: extreme gradient boosting.

Model performance across the five folds was evaluated using the mean absolute error (MAE), as shown in Figure 8. The mean MAE for each model was as follows: LR = 1.454, RF = 1.434, SVM = 1.673, and XGB = 1.716, with LR and RF demonstrating comparable performance. MAE represents the average absolute difference between the predicted and actual TUG test results, measured in seconds. For reference, the TUG test typically takes 8–12 s for individuals with normal physical function, whereas patients with impaired function may require more than 20 s. Thus, an MAE of approximately 1.4 s represents a relatively small proportion of the typical TUG test duration, indicating reasonable accuracy, particularly in patients with moderate to severe impairments. These results suggest that the models can provide meaningful estimates of physical function based on the input features, although further refinement is warranted to improve precision.

Considering the high multicollinearity, we opted to use only PVV for the final model development. RF was considered unsuitable due to the lack of depth in the developing trees, while LR was suitable for development with PVV.

### 3.6. Model Development with PVV

**Figure 9:**
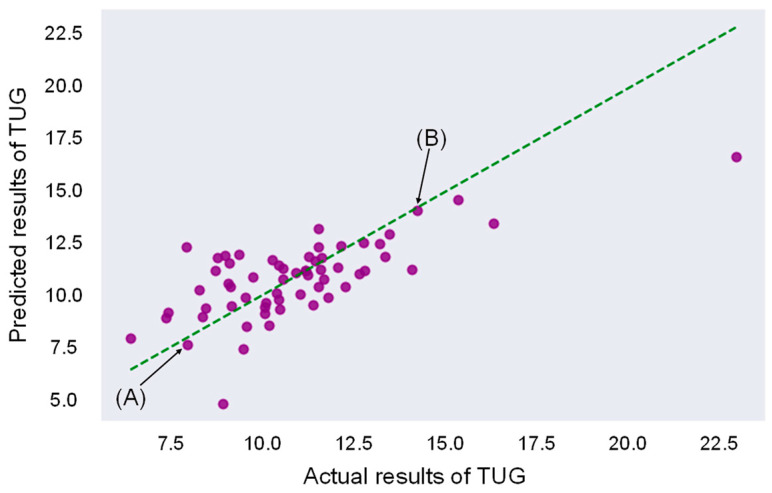
Scatter plot illustrating the predictions from five-fold cross-validation with the actual TUG results and presentation of representative cases. The MAE was 1.322, with a correlation coefficient of 0.713 between the predicted values and the actual values. Dots: respective cases, Line: An ideal situation where predictions and actual measurements match. Predicted results of TUG: predicted results of TUG by linear regression model; Actual results of TUG: measured results of TUG.

**Figure 10:**
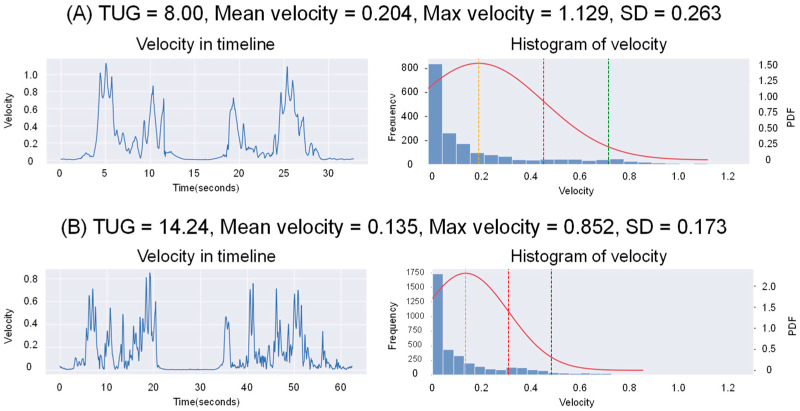
Details of cases (**A**,**B**) from Figure 9. Time series changes in the velocity in the xyz-direction (left) and normal curves based on the distribution, mean, and standard deviation (SD) of the velocity in the xyz-direction. The dotted line: mean velocity (yellow), +1SD (red), +2SD (green). PDF: Probability Density Function.

Using PVV, an LR model was developed to predict the results of TUG. The model’s performance was evaluated using five-fold cross-validation. The MAE and Pearson’s r of each fold are shown in Table 3, and the scatter plot of each fold shows the relationship between each training and validation sets in Supplementary Figure S1. The scatter plot shows the relationship between the predicted and actual results of TUG across all validation sets in Figure 9, with a mean MAE of 1.322 and Pearson’s correlation of r = 0.713 for the predicted TUG scores. Velocity plots over time and velocity distribution with a Gaussian curve are shown for two representative cases in Figure 10. Similar content is provided for two outlier cases in Supplementary Figures S2 and S3.

### 3.7. Model Development with xyz_SD_Vel_Standing

To explore additional features, we selected xyz_SD_Vel_Standing, which, as shown in Table 2, was identified as the second most informative feature using Lasso regression, and developed a separate LR model. The performance of this model was evaluated using five-fold cross-validation. A scatter plot showing the relationship between the predicted and actual TUG test results across all validation sets is shown in Supplementary Figure S4. This model achieved a mean MAE of 1.374 and Pearson’s correlation coefficient of r = 0.691 for the predicted TUG scores.

## 4. Discussion

We developed an LR model to predict the results of objective physical function assessments, specifically the TUG test. IMU data were collected from 61 patients with cancer during movements replicating a series of actions in a consultation room. We evaluated the velocity features from the 23 keypoints, identifying the pelvis as the most informative. From the pelvis keypoint data, we engineered 120 features related to the velocity and acceleration data. After performing feature selection and model comparison using Lasso regression in five-fold cross-validation, PVV was selected as the final feature for the LR model. This LR model with PVV achieved a correlation of 0.713 and MAE of 1.322. Higher PVV during the series of movements was associated with shorter TUG test results.

In this study, we focused on pelvis keypoints. A previous study showed high accuracy in predicting the SPPB and TUG results using timeline data from a single sensor attached to the right hip [42], where the participants wore the sensor for 2 weeks, capturing data throughout their daily activities. Although the duration of data collection differed from our study, where we recorded common daily actions, such as sitting, standing, and slow walking, focusing on pelvis keypoints appears reasonable for effectively capturing these movement features.

Variability in gait parameters has been a significant focus of previous studies on physical function. The coefficient of variation (*CoV*), a parameter used to measure variability, is calculated using the mean and *SD* as follows:

CoV = SD/mean

One study found that the CoV of step length and gait speed was significantly higher in survivors of breast cancer compared with controls without a cancer history in a zero-walkway setting (ProtoKinetics LLC, Havertown, PA, USA) [43]. Similarly, patients with chronic pulmonary disease exhibited a significantly higher CoV in stride time than healthy participants in treadmill walking [44]. These findings suggest that increased variability in gait parameters is associated with lower physical function in patients with a history of cancer or other chronic diseases.

In contrast, a study exploring the differences between laboratory and daily walking speeds compared the predictability of prefrailty using the mean, maximum, and SD of daily walking speed (DWS) [45]. DWS was recorded via a smartphone global positioning system application that automatically recorded walking speeds during daily activities over 1 month. The SD of daily walking speed (DWSsd) was calculated from over 50 walking measurements per participant, which demonstrated a higher area under the receiver operative curve of 0.615 compared to the mean and maximum DWS. A DWSsd cutoff value of less than 0.25 m/s was identified. This result aligns with our study: as the PVV decreased, the TUG test results increased. In summary, variability in gait parameters such as SD and CoV is associated with physical function assessment.

This study demonstrated that a higher PVV, which indicates greater variability in velocity, was associated with shorter TUG test times and potentially better physical function. Although there are several possible interpretations for this finding, we hypothesized that a higher PVV reflects a greater physical function reserve. In general, human movement is not performed at a constant speed; movement typically begins at a slower pace, reaches a peak speed in the middle, and then slows toward the end. Individuals with greater vitality and physical function capacity tend to achieve higher peak velocities during exercise, resulting in a greater amplitude between their maximum and minimum velocities. In addition, PVV may serve as a proxy for motor control ability, as individuals with higher physical functioning not only achieve higher speeds, but also demonstrate greater efficiency in controlling their movements. A higher PVV may reflect the ability to execute large changes in velocity smoothly and efficiently, suggesting a stronger relationship with physical function than peak velocity alone. However, further validation is required to confirm these findings and their broader implications.

In our study, PVV was selected as the final feature. Our findings suggest that the pelvis keypoint could be effective for developing AI-based assessments of physical functions from in-clinic-like movement videos, which is central to our concept. To our knowledge, no previous study has explored the possibility of objectively predicting physical function assessments from movements in a consultation room. Additionally, our findings may address the “black box” issue commonly associated with AI systems by identifying keypoints and features of interest.

While the model using xyz_SD_Vel_Standing demonstrated an MAE comparable to that using PVV, the lower correlation coefficient suggests that xyz_SD_Vel_Standing may not consistently capture the relationship with the TUG test results across the dataset. This reinforces the robustness and predictive strength of PVV as a primary feature of this study. Nonetheless, the performance of xyz_SD_Vel_Standing highlights its potential as a viable alternative, indicating that further exploration of additional features or combinations of features can enhance the overall performance of the model. Future studies should focus on evaluating these alternatives, particularly as larger datasets become available, to better understand the interplay between feature selection and predictive accuracy.

Building on these findings, our study demonstrated that the use of IMU data from the lumbar region alone was sufficient to measure the PVV, eliminating the need for a full-body IMU setup. Compared with previous studies on AI or other computational models for physical function assessment, this simplification has significant implications for the practical application of our system in clinical settings [46]. The ability to focus on a single region not only reduces the complexity and cost of the system, but also makes it accessible and easier to implement in routine practice. In addition, this approach paves the way for future developments where commonly available devices, such as smartphones, can measure PVV by simply being placed in a pocket near the waist. This direction aligns with broader trends in healthcare innovation observed in the post-COVID-19 era, with an increased emphasis on leveraging readily available, cost-effective technologies to enable remote monitoring and decentralized care [47]. By reducing dependency on specialized equipment and creating more adaptable solutions, this approach supports the paradigm shift towards more resilient and scalable healthcare systems. Such advancements can further improve the feasibility and scalability of AI-based physical function assessment systems, enabling their widespread use in diverse clinical settings. These results highlight the potential of our system to bridge the gap between advanced AI-based analytics and real-world clinical applications by addressing practical limitations, while maintaining the rigor of objective physical function assessments.

This study has some limitations. First, the dataset comprised only participants with a PS of 0 or 1, resulting in an imbalanced distribution of the SPPB results. This is likely because participants with poorer physical functions were excluded or withdrew consent, as recruitment occurred during their first hospitalization for chemotherapy, with data collection during subsequent hospitalizations, which posed inevitable logistical challenges in arranging space for objective physical function assessment and IMU data collection. Whether this model can be applied to vulnerable patients who cannot be administered chemotherapy needs to be validated in the future. Second, the sample size was small (61 cases), and external validation was not conducted. We acknowledge that the small sample size is a significant limitation of this study. In addition, while we ensured that the participants used for validation in each fold were not included in the training set during cross-validation, we did not allocate a separate test set before performing the cross-validation. This decision was made because of the limited number of cases, as reserving a dedicated test set would have further reduced the amount of data available for model training and validation, potentially compromising the robustness of the analysis. This limitation will be addressed in future studies, as larger and more diverse datasets become available. Third, the limited sample size informed our choice to use a linear regression (LR) model instead of more complex machine learning models, such as neural networks, because these models carry a higher risk of overfitting when applied to small datasets. However, as we expand our dataset for future studies, exploring the application of more complex models to capture potential nonlinear relationships is a key area of focus. Fourth, most participants were male, reflecting the epidemiological characteristics of head, neck, and esophageal cancers. Fifth, it is unclear how accurately the movements replicating a series of actions in a consultation room reflect actual clinical movements, as consulting room sizes and the duration of seated time can vary across medical institutions. Sixth, while the LR model using PVV indicated a trend towards shorter TUG times with increasing PVV, its performance was not sufficient (Supplementary Figures S2 and S3). Further development of an AI-based physical function assessment system should consider enhancing performance by exploring video-specific features or further feature engineering.

## 5. Future Directions

This study provides a foundation for the further development and application of the proposed model, with several potential directions for future research. First, we plan to expand the target population, aiming to include patients with different types of cancer to validate the applicability of the model to a broader range of oncology populations. Then, we plan to expand this study to include patients with non-cancerous conditions and, finally, individuals who visit hospitals for preventive purposes. A study involving other types of cancer is already in the planning stages. Second, while this study focused on features extracted solely from IMU data, future work will explore the integration of additional features to improve the model performance. One promising avenue is the incorporation of features derived from motion capture videos, which can provide more detailed insights into movement patterns. In addition, speech features such as tone or pitch can serve as valuable inputs for refining the model. Finally, a longitudinal assessment is important. Collecting data at multiple time points could allow the prediction of future changes in physical function, such as TUG performance over time. In addition, collecting data before and after therapeutic interventions may allow the model to predict whether treatment is likely to improve a patient’s ADL, thereby providing valuable support for clinical decision-making. These steps will further refine the model and increase its utility in both clinical and preventive settings.

## 6. Conclusions

This study demonstrated that PVV is a valuable feature for predicting TUG test results, with the LR model achieving a Pearson’s r of 0.713. However, further data collection and analysis are required to improve and validate the model’s accuracy, leading to the development of an AI-based physical function assessment system based on actual in-clinic movements.

## Figures and Tables

**Table 1:** Patient cohort characteristics (n = 61).

Characteristics	Values
Age, median (IQR)	71 (65–75)
Sex	
Male	48
Female	13
BMI, mean (minimum–maximum)	21.6 (14.7–29.7)
ECOG PS	
0	33
1	28
Cancer type	
Esophageal cancer	44
Head and neck cancer	13
Gastroesophageal junction cancer	2
others	2
Clinical stage (UICC 8th)	
I	10
II	3
III	22
IV	17
Recurrence	9
Type of chemotherapy	
Induction chemotherapy	44
Neoadjuvant or induction chemotherapy	11
Palliative chemotherapy	6

Abbreviations: IQR, Interquartile Range; BMI, Body Mass Index; ECOG PS, Eastern Cooperative Oncology Group Performance Status, UICC: Union for International Cancer Control.

**Table 2:** Frequency analysis of extracted pelvic features by Lasso regression in five-fold cross-validation.

Feature	Count
xyz_SD_Vel_Throughout	5
xyz _SD_Vel_Standing	3
z_Min_Vel_Standing	1
xyz_Max_Vel_Throughout	1
z_Max_Acc_Standing	1
xyz_Mean_Acc_Standing_up	1
xy_Min_Acc_Sitting_down	1
xyz_Mean_Vel_Standing_up	1
xyz_Max_Vel_Standing	1

xyz: xyz-direction, z: z-axis, xy: xy-plane, SD: standard deviation, Min: minimum, Max: maximum, Vel: velocity, Acc: acceleration.

**Table 3:** The metrics of each fold in five-fold cross-validation of LR model using PVV.

Fold	1	2	3	4	5
MAE	1.118	1.311	1.367	1.720	1.096
Pearson’s r	0.738	0.544	0.713	0.866	0.704

MAE; mean absolute error.

## Data Availability

The original contributions presented in this study are included in the article/Supplementary Material. Further inquiries can be directed to the corresponding authors.

## Supplementary Materials

The following supporting information can be downloaded from https://www.mdpi.com/article/10.3390/bioengineering11121232/s1: Figure S1. The scatter plot of each fold of TUG, showing each training set result and validation set result. Figure S2. Scatter plot of prediction in five-fold cross-validation with actual TUG results and presentation of outlier cases. Figure S3. Details of cases C and D in Supplementary Figure S1. Figure S4. Scatter plot illustrating the predictions from five-fold cross-validation using xyz_SD_Vel_Standing with the actual TUG results. Video S1. The actual video of movements replicating a series of actions in a consultation room.
