# Supplementary material for: Analysis of Inertial Measurement Unit Data for an AI-Based Physical Function Assessment System Using In-Clinic-like Movements
Source: Bioengineering (Basel). 2024 Dec 5;11(12):1232. doi: 10.3390/bioengineering11121232 (PMC11673146; doi:10.3390/bioengineering11121232)
Supplement: Supplementary file 1 [file bioengineering-11-01232-s001.zip › Supplementary Files/Supplementarry Information Bioengineering_IMU_ProofReading.pdf]

Supplementary information for
Analysis of Inertial Measurement Unit Data for an AI-based Physical Function Assessment System using In-Clinic-like Movements

Nobuji Kouno, Satoshi Takahashi, Ken Takasawa, Masaaki Komatsu, Naoaki Ishiguro, Katsuji Takeda, Ayumu Matsuoka, Maiko Fujimori, Ken Kato, Kazutaka Obama, Ryuji Hamamoto

The file contains
Supplementary Figures S1 – S4

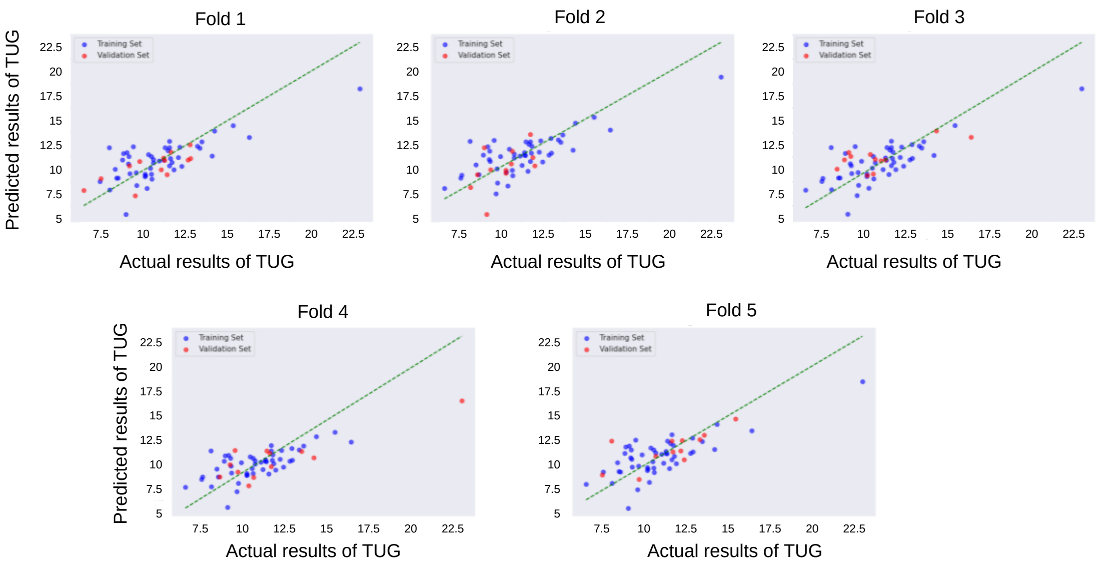

Figure S1. The scatter plot of each fold TUG, showing each training set and validation set results. The metrics for each fold are as follows: Fold1, mean absolute error (MAE) = 1.118, Pearson's r = 0.738. Fold2, MAE = 1.311, Pearson's r = 0.544. Fold3, MAE = 1.367, Pearson's r = 0.713. Fold4, MAE = 1.720, Pearson's r = 0.866. Fold5, MAE = 1.096, Pearson's r =0.704: Timed up and go test, Predicted results of TUG: predicted results of TUG by linear regression model, Actual results of TUG: measured results of TUG.

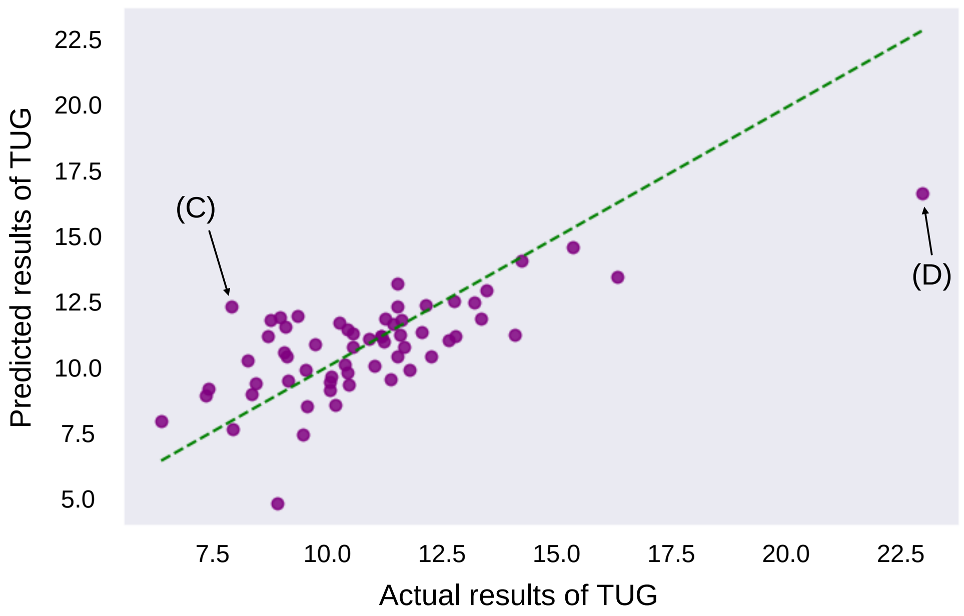

Figure S2. Scatter plot of prediction in five-fold cross-validation with actual results of TUG, and presentation outlier cases. Dots: respective cases, Line: An ideal situation where predictions and actual measurements match.

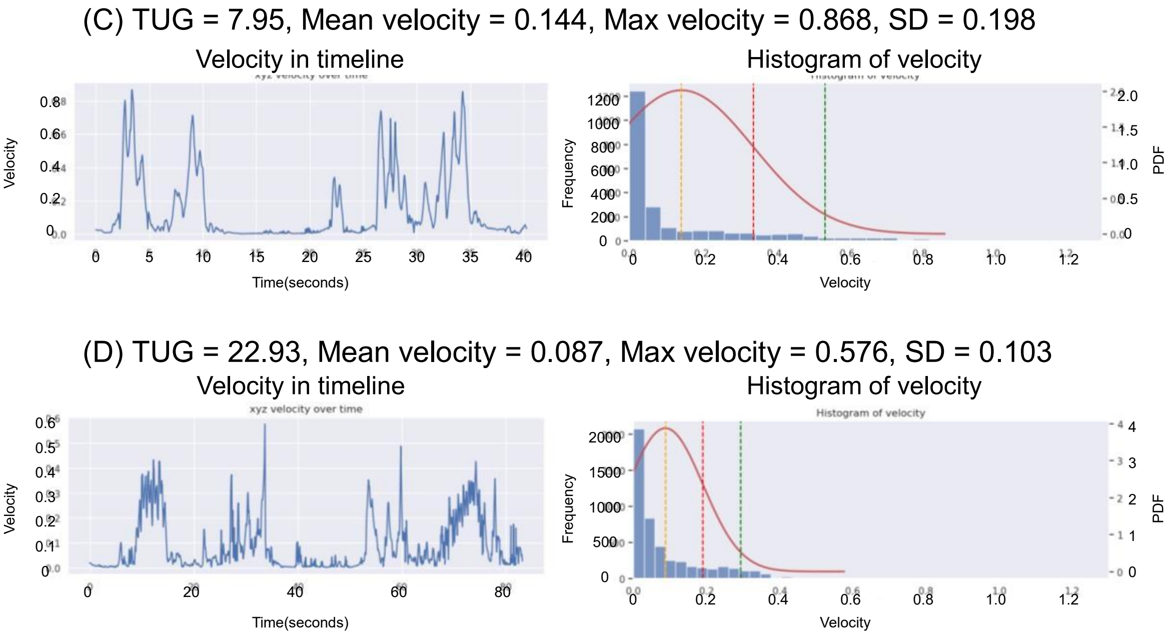

Figure S3. Details of cases C and D in **Supplementary Figure S2**. Time series changes of the velocity in the xyz-direction (left) and normal curves based on the distribution, mean and standard deviation (SD) of the velocity in the xyz-direction. dotted line: Mean velocity (yellow), +1SD (red), +2SD (green). PDF: Probability Density Function.

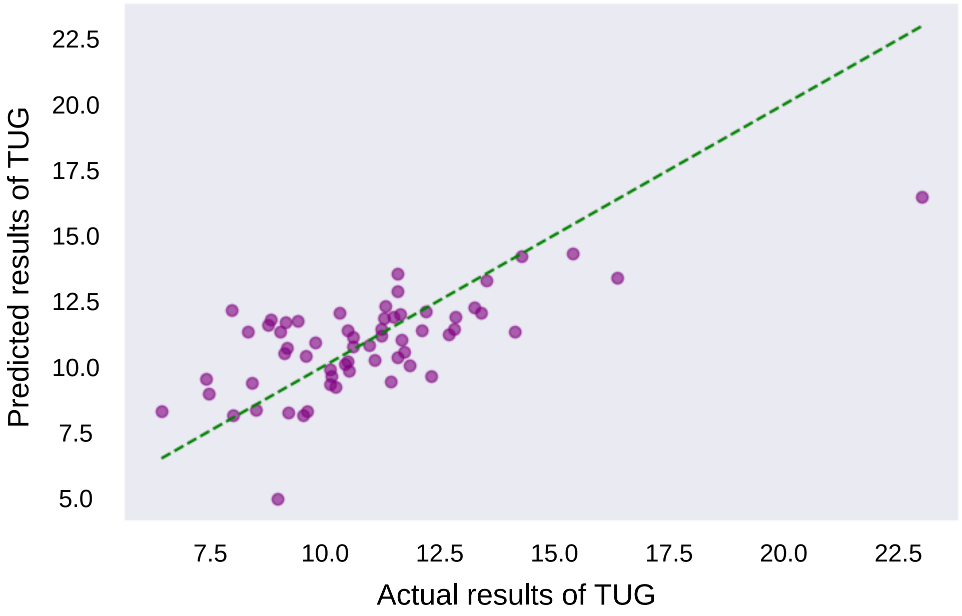

Figure S4. Scatter plot illustrating the predictions from five-fold cross-validation using xyz_SD_Vel_Standing with the actual TUG results. The MAE was 1.374, with a correlation coefficient of 0.691 between the predicted values and the actual values.
